# A Comparative Analysis of Laser Additive Manufacturing of High Layer Thickness Pure Ti and Inconel 718 Alloy Materials Using Finite Element Method

**DOI:** 10.3390/ma14040876

**Published:** 2021-02-12

**Authors:** Sapam Ningthemba Singh, Sohini Chowdhury, Yadaiah Nirsanametla, Anil Kumar Deepati, Chander Prakash, Sunpreet Singh, Linda Yongling Wu, Hongyu Y. Zheng, Catalin Pruncu

**Affiliations:** 1 Department of Mechanical Engineering, North Eastern Regional Institute of Science and Technology, Nirjuli 791109, India; sapamthemba@gmail.com (S.N.S.); sohinidme@gmail.com (S.C.); 2 Department of Mechanical Engineering, National Institute of Technology Silchar, Silchar 788010, India; 3 College of Applied Industrial Technology, Jazan University, Jizan 45142, Saudi Arabia; flytoanil@gmail.com; 4 School of Mechanical Engineering, Lovely Professional University, Phagwara 144411, India; 5 Department of Mechanical Engineering, Shandong University of Technology, Zibo 255000, China; ylwu06@sdut.edu.cn (L.Y.W.), zhenghongyu@sdut.edu.cn (H.Y.Z.); 6 Mechanical Engineering, National University of Singapore, Singapore 119077, Singapore; snprt.singh@gmail.com; 7 Mechanical Engineering, Imperial College, Exhibition Rd., London SW7 2AZ, UK; 8 Design, Manufacturing & Engineering Management, University of Strathclyde, Glasgow G1 1XJ, UK

**Keywords:** laser additive manufacturing, Inconel 718, pure Ti, finite element modeling, melt-pool formation

## Abstract

Investigation of the selective laser melting (SLM) process, using finite element method, to understand the influences of laser power and scanning speed on the heat flow and melt-pool dimensions is a challenging task. Most of the existing studies are focused on the study of thin layer thickness and comparative study of same materials under different manufacturing conditions. The present work is focused on comparative analysis of thermal cycles and complex melt-pool behavior of a high layer thickness multi-layer laser additive manufacturing (LAM) of pure Titanium (Ti) and Inconel 718. A transient 3D finite-element model is developed to perform a quantitative comparative study on two materials to examine the temperature distribution and disparities in melt-pool behaviours under similar processing conditions. It is observed that the layers are properly melted and sintered for the considered process parameters. The temperature and melt-pool increases as laser power move in the same layer and when new layers are added. The same is observed when the laser power increases, and opposite is observed for increasing scanning speed while keeping other parameters constant. It is also found that Inconel 718 alloy has a higher maximum temperature than Ti material for the same process parameter and hence higher melt-pool dimensions.

## 1. Introduction

Additive manufacturing (AM) has sustained and advanced as a manufacturing process from rapid prototyping (RP). Earlier, AM was mainly used for prototyping, but with due time and thanks to the contributions of many researchers, now, it can be employed in the commercial manufacturing of aircraft parts, prosthesis, spacecraft parts, high strength military equipment, automobile parts, etc. Against the conventional machining principle where the product is manufactured by removing unwanted materials from the blank, AM manufactures the components from scratch by adding materials in a layer by layer order directly from the sliced CAD model. AM offers various advantages such as printing multi-material parts, manufacturing of functionally graded materials, the ability to print highly customized biomedical parts, a little change in tools and machine layout for new designs, etc. The ability to manufacture complex shapes using lightweight materials by AM is another leverage that AM has over the conventional machining processes in the aerospace industry, as lightweight materials can be manufactured into complex shapes without losing strength using AM. Despite these recent advances, it remains very costly, low efficiency in terms of material handling, process costs, overall machine acquisition, and maintenance costs. One of the most critical factors in optimizing these bottlenecks is the slow build time. Slow manufacturing time can be improved if the AM of high layer thickness materials is achieved. Hence, a clear understanding of the process is required.

In the aerospace industries, safety and capability are the two main concerns besides the cost involved. In such cases, Titanium (Ti) is the preferred material because of its desired properties being relatively lightweight yet high in strength, higher operating temperature range, ability to resist corrosion, and the ability to adapt to other composites [1]. However, in traditional manufacturing processes, the usage of Ti is limited as the cost is high as compared to other materials. With AM technology’s advancement, AM of Ti is drawing more tractions [2,3,4,5]. Apart from the material-specific problems such as high surface roughness, irregular and concentrated residual stresses; long manufacturing lead time and slow build rate are also significant hindrances in adopting laser additive manufacturing (LAM) processes in aerospace industries. Inconel 718 alloy material has its applications in aerospace industries as well as in biomedical applications. Ti and Inconel 718 alloys are used in manufacturing aircraft fan blades, turbine discs, pressure compressors, etc. Some of the major applications in biomedical industries are surgical, dental, orthopaedic devices, and stents [6,7]. With the ever-increasing applications of these two materials in aerospace and biomedical applications, both face unique yet identical problems while fabricating. There are many process parameters in a laser-based AM process. Laser power, scanning speed, hatching distance, layer thickness, and scanning pattern are important process parameters in a laser-based AM process that can be adjusted to optimize the manufactured parts’ properties [8,9,10,11]. Jang et al. reported that laser power is the most influential parameter on surface roughness, hardness, and density [12]. However, Calignano et al. [13] reported that scanning speed has the highest impact on the surface roughness of the fabricated components. The contradicting reports may be due to the difference in materials and the use of different machines. The Taguchi method, response surface method (RSM), and artificial neural networks (ANNs) are the popular optimization methods used to optimize the process parameters in a laser-based AM process. The Taguchi method has been applied to optimize the AM process for various materials including Ti, Inconel, SS316L, AlSi10Mg, etc. [12,13,14,15,16,17,18]. Similarly, the RSM method has been used to optimize the major process parameters i.e., laser power, scanning speed, scanning pattern, etc. [19,20,21]. Fotovvati et al. reported that layer thickness is one of the important factors regarding surface roughness of Ti-6Al-4V alloy in a laser-based powder bed fusion AM process. Using the Taguchi method, the authors also recommended using the highest energy density i.e., high laser power and low scanning speed. Laser power and hatching space are the most significant parameters for micro-hardness and density of the fabricated parts [22]. Khorasani et al. implemented ANN-based models and found that the heat treatment conditions play a major role in the final surface roughness of the manufactured parts [23].

Several research works have been reported with regards to the numerical modeling and finite element-based analysis of the AM process. The basic model consists of a heat source applied to the layers where analysis of the heat interaction is observed based on the finite element modeling [24,25]. A single layer model is very helpful in terms of initial modeling and validation of the model, extending into a multi-layer model to replicate the real-world scenario. From the analytical point of view, the thermal cycle repeats after a certain number of layers. Keeping the computational cost and time in mind, a model should be restricted to a limit where there is a significant influence on the thermal cycle.

A simple thermal model was analysed by Alexander et al. that considered the heat source model, thermal conductivity, and other parameters [26]. Other researchers have also proposed their own models based on different heat source models [27,28,29]. There are different heat source models such as the Gaussian distribution heat source model, cone type heat source models, Goldak’s ellipsoidal model, logarithmic decay model, etc. [8,30,31,32,33,34,35,36,37,38]. Li et al. analysed the residual stress during the selective laser melting (SLM) of 355 steel using the Gaussian volumetric model and found that the model can predict the stress distribution accurately [31]. The use of the Gaussian distributed heat source model is further supported by Teixeira et al. in their numerical simulation of the TIG welding process using the Gaussian distributed heat source model [34]. Wei et al. performed a thermos-mechanical analysis on the hot cracking of laser welding of lap joints by adopting the Gaussian model, but they extended the model by taking the linear decay in the Z direction [36]. Soldner et al. extended numerical models to account for crystallization of the printed parts in the selective laser sintering (SLS) of PA12 materials and found that the crystallization occurs even during the manufacturing process. They also found that the temperature distribution is dependent on the geometry orientation of the parts [39].

Wu et al. found that the heat flow and melt-pool dimensions have a great impact on the final product’s residual stress as well as on other mechanical properties during the SLM of AlSi10Mg [40]. The melt-pool size increases as the laser scanning continues and reaches a steady state. Higher beam intensity and slower beam speed have faster melt-pool evolution, while lower intensity with faster beam speed has the opposite effect. A larger melt pool is observed in the subsequent layers. Similar patterns are also observed for other materials. Another study was carried out by Hu et al. on multi-layer SLM of AlSi10Mg material. The gradual increase of temperature and melt-pool were reported with the addition of new layers, which helped in obtaining fine grains for low energy input [41]. A similar numerical study was carried out by Khan et al. [42]. An experimental investigation was performed to measure the residual stress of SLM-manufactured AISI 316L samples. On the top surfaces, higher residual stresses in the scan direction were reported than in the downward direction. However, in the lateral surface, the residual stresses are higher in the perpendicular direction than that of the scan direction. This is due to the temperature gradient and cooling down of the substrate [43]. In another study, it was found that Ti-6A1-4V experiences more residual stress than Inconel 718 material [44]. However, little research is being pursued on a high layer thickness material. As opposed to the general concept, Jingjing et al. found that layer thickness has very little effect on the melt-pool dimensions for a Ti-6Al-4V material by the SLM process [45]. Most of the reported studies concentrated on the very thin layer thickness, which is a limiting factor on the final material addition rate and hence the manufacturing time.

Song et al. presented the effects of scanning patterns on residual stress in an SLM process of Ti-6Al-4V. Even though there is an influence of scanning patterns on the residual stress, no significant change in the melt-pool size was observed [46]. Yang and Wang presented a case study of a repair process by direct laser fabrication (DLF) in ABAQUS software to study the influence of non-linear temperature-dependent properties in a pure nickel material on temperature distribution [47]. It is obvious that the highest peak temperature value is at the point or region where the laser is applied while the second peak value is at the border of the current layer and the previous layer, and the third peak value is at the border of the previous layer and second previous layer. There is a sudden rise in the thermal conductivity and heat transfer at the point where the phase change takes place. Direct metal laser fabrication (DMLF) is designed specifically for metal parts. However, it can be used for non-metal and polymer materials as well [48].

A high layer thickness of an AM process comes with some inherent problems such as an increase in surface roughness, a higher tendency to dimensional distortions and, improper melting. Martínez et al. found that the scan strategy, thermal cycle, and residual stresses affect, in part, distortion, especially on parts where a high strength to weight ratio is considered [49]. Nadammal et al. found that the rotational and alternate scanning pattern results in the least residual stress and distortion [50]. A study by Ventola et al. observed that additively manufactured rough surfaces have enhanced heat transfer and can be used in electronics cooling [51]. Yang et al. investigated the vertical surface roughness of AlSi10Mg parts manufactured by the SLM process instead of the common research on horizontal surface roughness. The authors determined that the surface roughness can be reduced by about 70% from 15 µm to 4 µm by using the energy density approach [52]. Such inherent problems like the surface roughness can be used to our advantage in applications where enhanced heat transfer is required without much concern regarding the surface roughness of the materials. In such areas, the use of a high layer thickness will improve the manufacturing time.

Ding et al. performed a numerical analysis of heat transfer along with the fluid flow analysis for different laser scanning patterns in an SLM process. In a point laser exposure, a wide melt-pool was observed. In addition to this, there was a longer processing time with multiple thermal cycles, which resulted in finer grains [53]. Another advantage that AM offers is the ability to provide onsite demand and manufacturing of spare parts, which will reduce the manufacturing lead time (MLT), inventory, and transportation cost [54]. By adopting onsite demand, the US Navy was able to reduce the cost by 30% and increase on-demand part production [55,56]. A comparative study of samples is reported based on different manufacturing processes. Chastand et al. investigated different fatigues of Ti-6Al-4V samples produced by electron beam melting (EBM) and the SLM process. It is observed that surface defects have a high impact, followed by un-melted zones and small internal defects [57]. A recent study found that the short-time creep resistance of Inconel 718 manufactured by the SLM process is comparatively the same as that of manufactured by forging process. However, it is far below when compared to a sample manufactured by the casting method [58]. Without denying the importance of these investigations, most of the research concentrates on the comparison of the same material manufactured by different manufacturing processes. Limited information is available that shows a comparative study of two materials viz. Ti and Inconel 718 in terms of temperature distribution and different melt-pool dimensions under the same processing conditions manufactured by the LAM process. Such a study will help in determining which exact material to use in specific conditions or even use both the materials.

However, the AM process faces some major problems that are hindering the mass adoption of this technology on commercial scales. Another major issue is the limited availability of high performance yet light in weight materials. Some of the materials that are available are difficult to process using the AM technique, or it exhibits poor mechanical properties even if it is possible for AM to process those materials. Given the importance of LAM in aerospace and biomedical applications combined with the ever-expanding use of Ti material and Inconel 718 alloy, these two materials are considered in the present study. Another major hindrance is the high manufacturing lead time, which is largely because of the very thin layer thickness. The layer thickness of the most SLM process mentioned in this section is below 100 µm. Manufacturing a whole part with this scale of layer thickness will take a long time to complete. Adopting a higher layer thickness will substantially reduce the MLT of the item. Additionally, limited information is available on the interaction and process information for different materials for additively manufactured parts of high layer thickness materials. Therefore, the present study is focused on the investigation of thermal interaction and change in melt-pool dimensions due to thermal interactions in between the layers and substrates in a high layer thickness multi-layer LAM process of pure Ti and Inconel 718 materials and comparatively analyze the results.

The present paper shows the comparative numerical investigation of laser-based additive manufacturing of high layer thickness materials. A Gaussian distributed disc heat source model is used as the laser heat source model. To simulate the layer addition, the element death and birth feature is employed. It also investigates the effects of laser power and laser scanning speed on the heat flow and melt-pool dimensions. Based on the observed results, it is found that LAM of high layer thickness materials is possible with sufficient process parameters, well under the capability of currently available LAM machines.

## 2. Numerical Modeling

**Figure 1:**
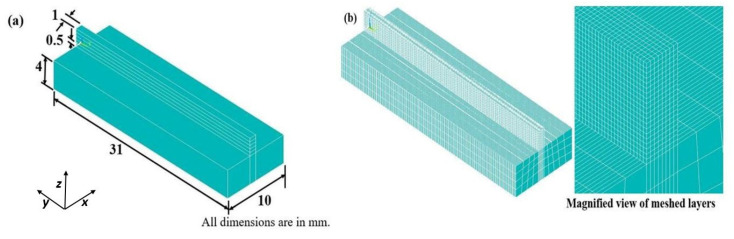
(**a**) Geometry of the FE model and (**b**) FE model of a five-layer selective laser melting (SLM) for Ti and Inconel 718 alloy material.

A finite element-based numerical model is developed for transient thermal analysis using ANSYS (APDL) 14.5 developed by the ANSYS Inc. Canonsburg, PA, USA. The SOLID70 element having a single degree of freedom (DOF) is chosen during finite element (FE) thermal modeling. Figure 1 shows the FE model of Ti and Inconel 718 materials. The substrate size is 10 mm × 31 mm × 4 mm with a layer size of 1 mm × 31 mm × 0.5 mm. Figure 1b shows the meshed FE model along with a magnified view. Most of the heat transfer and interaction will take place in and around the layers. With an aim to reduce the computational time and cost, fine-mesh is adopted for the layers, and coarse-mesh is adopted for the substrates. To mimic the addition of new layers on top of existing layers, the element birth and death feature is employed. Even though five layers are already modelled, the upper four layers are marked as death elements and do not take part in any physical and thermal interactions. After scanning of the first layer, practically a new powder layer is added, and the 2nd layer is marked as active while the other three upper layers are still marked as death elements. This process is continued until the scanning of the last layer (5th).

**Figure 2:**
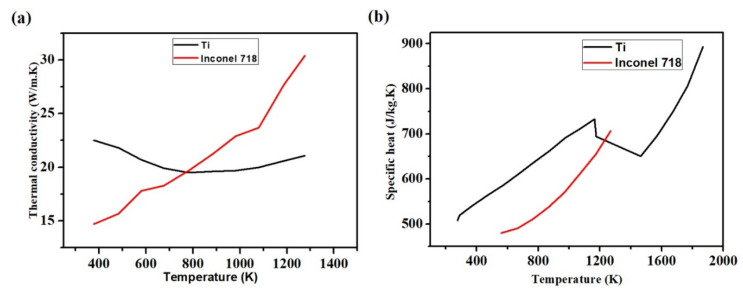
Temperature-dependent (**a**) thermal conductivity and (**b**) specific heats of Ti and Inconel 718 [44,59,60,61].

The process parameters employed during the LAM process for Inconel 718 and pure Ti are given in Table 1. The layer thickness is kept constant at 0.5 mm for both materials. Temperature-dependent physical material properties of Ti and Inconel 718 are given in Figure 2. The other material properties of the two materials are given in Table 2.

**Figure 3:**
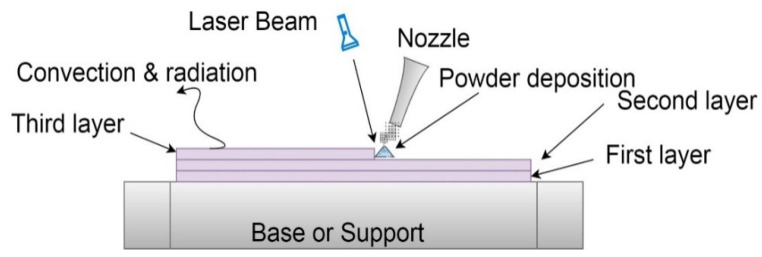
Schematic of boundary conditions of a multi-layered SLM process.

During the SLM process, the energy from the laser beam strikes the metal powders, which melts, and the energy is transferred to subsequent layers. By repeating this process, some layers are subjected to thermal changes several times, thus resulting in phase transformation, grain growth, a final product with non-uniform grain size, shape, and thermal distribution. Moreover, during the SLM process, the object is subjected to convection and radiation heat losses. Figure 3 presents a schematic of thermal boundary constraints of a multi-layered SLM process.

A finite 3D model is developed for the laser additive manufacturing process using ANSYS software to determine the non-linearity and temperature variations with respect to time. The 3D heat conduction equation (transient) is represented by [34]

(1) $$k\left(\frac{\partial^{2}T}{\partial x^{2}}+\frac{\partial^{2}T}{\partial y^{2}}+\frac{\partial^{2}T}{\partial z^{2}}\right)+\dot{\dot{Q}=\rho c_{p}\left(\frac{\partial T}{\partial t}\right)}$$

where *k*, $\;\dot{Q}$, *ν*, *C_p_*, *T*, and *ρ* are thermal conductivity, rate of internal heat generation per unit volume, velocity vector, specific heat, temperature, and density of the material, respectively. The terms on equation 1 represent heat transfers in X, Y, and Z directions with laser heat power supplied for melting of the powder layers. The initial condition at the start of the operation is given by

(2) $$T\left(x,y,z\right){|}_{t=0}=T_{0}$$

where *T*_0_ represents ambient temperature (298 K i.e., 25 °C).

Most of the heat transfer takes place due to phase change, latent heat of conduction in and around the area where laser heat is applied, and melt-pool occurs. Convection and radiation heat transfer are assumed in all the exposed surfaces of the substrate. The natural boundary condition can be represented mathematically, which involves convective heat transfer and radiative heat transfer [64,65,66,67,68,69,70]:

(3) $$k\frac{\partial T}{\partial n}-q+q_{c}+q_{r}=0$$

where *q* is the input heat flux, *q_c_* and *q_r_* are heat loss due to convection and radiation respectively. The basic equation of convective and radiative heat transfer can be represented as:

(4) $$q_{c}=h\left(T-T_{0}\right)$$

(5) $$q_{r}=\sigma \epsilon \left(T^{4}-T_{0}^{4}\right)$$

where h is the convective heat transfer co-efficient, σ  is the Stephan–Boltzmann constant and ϵ is the emissivity, *T*_0_ and *T* are the initial temperature and instant temperature.

The Gaussian heat distribution model is the most common model used in a laser-based additive manufacturing process as the heat distribution, which follows Gaussian distribution. Gaussian distributed disc model is implemented as the heat source to mimic the laser heat source onto the powder layers from the laser source, which is mathematically represented as [32,33,71].

(6) $$q=\frac{3\eta P}{\pi r^{2}}exp(\frac{-3\left(x^{2}+y^{2}\right)}{r^{2}}),$$

where *q, η, P* and *r* represents the rate of laser heat flux, the efficiency of the laser beam absorption, absolute laser beam power, effective radius of the laser beam at the surface of the layer. *x* and *y* depict the position of the centre of the laser beam at a specific time and at the respective layer.

## 3. Results and Discussion

**Figure 4:**
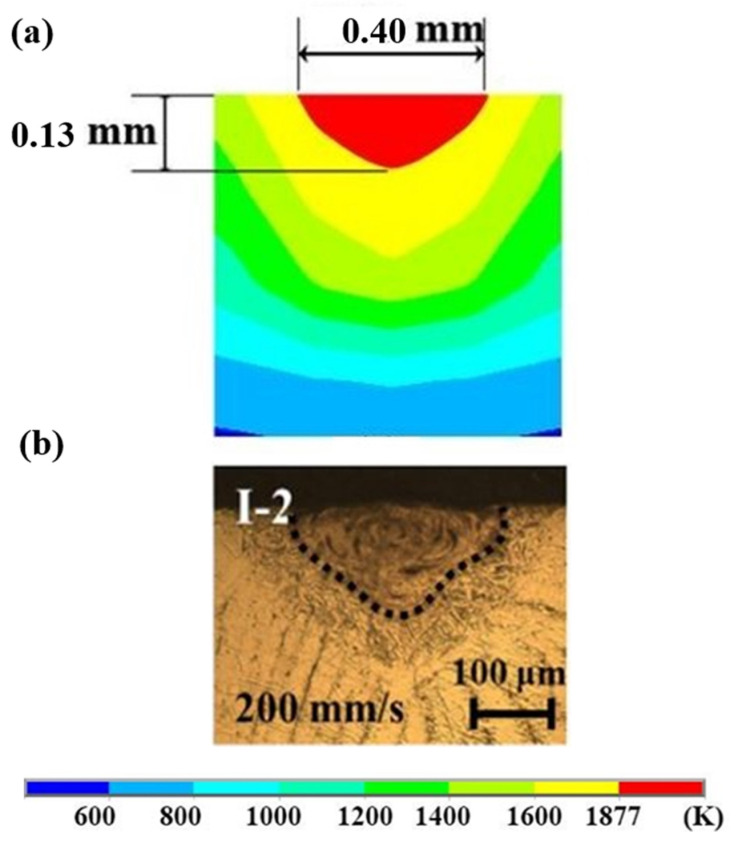
Comparison of (**a**) computed and (**b**) experimental melt-pool shape and size.

The numerical simulation is performed for Ti and Inconel 718, corresponding to the dataset in Table 1, and a detailed analysis of the LAM process is studied. The temperature behavior against time and nature of the melt-pool of Ti and Inconel 718 materials are studied separately. For different datasets, the comparative study of the two materials under the same process parameters are studied and analysed to determine the nature of transient heat transfer and temperature over time. The developed model has been validated with the experimental data available in the literature [45]. First, a single layer FE is developed for Ti-6Al-4V material and is compared with the experimental data available in the literature. As shown in Figure 4, the melt-pool dimensions of computed value are ~0.12 and ~0.13 mm for the experimental data. The pattern and data agree with the computed value and experimental value. The percentage difference in the melt-pool depth is estimated to be 5.38% which is under the acceptable range. This model is further extended into a multi-layer model with the material properties considered in the present paper.

### 3.1. Melt-Pool Evolution and Temperature Distribution

**Figure 5:**
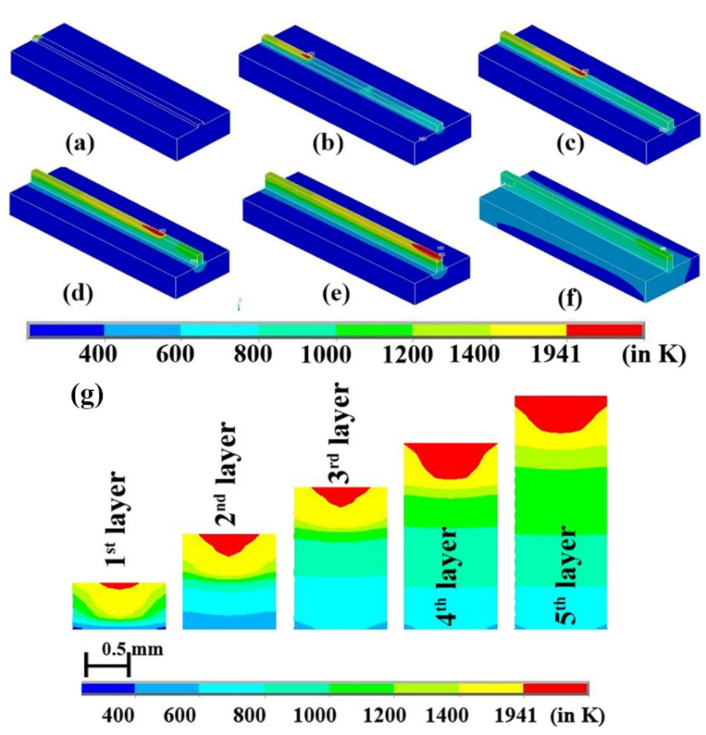
Laser movement and cooling over different layers (**a**–**f**) and changing melt-pool dimensions of laser scanning during a laser AM process of 5 layers for pure Ti for dataset#6 (**g**).

The melt-pool dimension increases as the laser power move along the direction of the laser scan. Moreover, the melt-pool size and length increased as the new layers are added. However, there are un-melted powders in some parts of the layer when laser power is 150 W and the laser velocity is 150 mm·s^−1^. This is possibly due to the applied laser power is not enough to melt the whole layer thickness. Due to this, the specific dataset is not mentioned in Table 1. Figure 5 represents the 3D view of laser movement along the different layers and evolution of the melt pool. Figure 5a shows the laser movement along the layer at the beginning of the 1st layer. Similarly, Figure 5b–e shows laser movement along the 2nd layer, at the middle of the 3rd layer, at the 4th layer, and at the end of the 5th layer. Figure 5f displays cooling after laser scanning is over for dataset#6 of Table 1. The cross-sectional view of melt-pool alteration is shown in Figure 5g, corresponding to different layers. Moreover, the cumulative effect of the increasing size and length of the melt-pool can be visually recognized in Figure 5g. The zone above 1941 K denotes part of the layer which is fully melted. The 1400–1941 K zone indicates the sintered area. The other zones do not take part either in melting or sintering.

In Figure 5g, it is observed that the melt-pool depth and width increased as the process continue and as layers are deposited on top of one another. The width increases from ~0.42 to ~0.62 mm from 1st layer to the 2nd layer and then to ~0.65 mm for the third, while, fourth and fifth layers have a width of 1 mm or higher. It was observed that the melt pool depth increased from ~0.088 to ~0.41 mm from the 1st layer to the 5th layer. The melt pool depth corresponding to the 2nd layer, 3rd layer, and 4th layer was determined to be ~0.21, ~0.24, and ~0.38 mm, respectively. At the beginning of the 1st layer, it is observed that the whole depth of the layer is not melted. This can be attributed to the fact that at the beginning of the process, the substrate’s temperature and layers are at ambient temperature, which prevents it from reaching the melting point. Preheating the powder materials may reduce the improper melting of layers at the beginning of the process. However, as the laser supply and heat transmission from melt pool to substrate continue, the overall temperature of the substrate and the layers increases that enables the laser focus area to attain higher maximum temperatures. With temperature rise, the melt-pool depth increases. This shows that the manufacturing of products by the LAM process is possible. From the 4th to 5th layer, there is a decrease in the rate of increasing melt-pool dimensions when compared to the other layers. This indicates the stagnation point where a further increase in the layers will not affect the melt-pool dimensions significantly. This phenomenon can be attributed to the fact that the temperature of the initial layers is nearing the approximate substrate temperature. However, the overall temperature of the substrate is increased and remains the principal heat transfer agent. Considering the high layer thickness, it will be of great interest if further research is undertaken for a sloped surface as performed by Xiang et al., where the slope’s quality is analysed. Such an analysis by considering different oblique angles under different process parameters will be of great interest as a higher layer thickness may produce more extreme results in increasing or decreasing the quality of slope on the surfaces [72].

**Figure 6:**
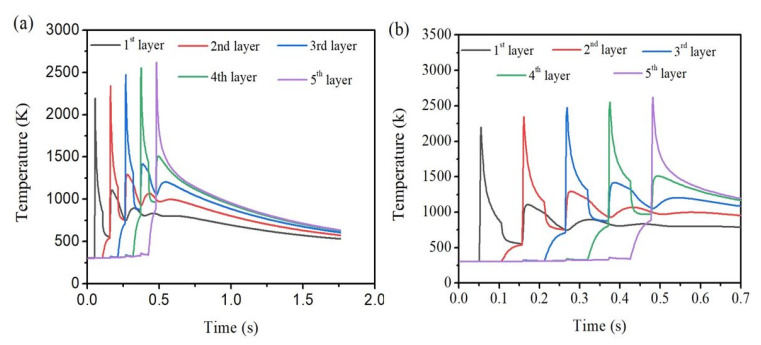
Time-temperature history (**a**) full view, (**b**) zoomed view considering only up to 0.7 s of scanning time, at the centre of each layer for dataset#6.

Figure 6 represents the time-temperature graph at the midpoint of each layer when power is 250 W and speed is 300 mm·s^−1^. The maximum temperature obtained at the middle of the 1st layer is 2195 K, 2342 K for the 2nd layer, 2475 K for the 3rd layer, 2552 K for the 4th layer, and 2622 K for the 5th layer. This confirms the general prediction that the maximum peak temperature increases with each increasing layer number. Thereby, the melt-pool size is increased, and it is above the melting temperature of the Ti material.

**Figure 7:**
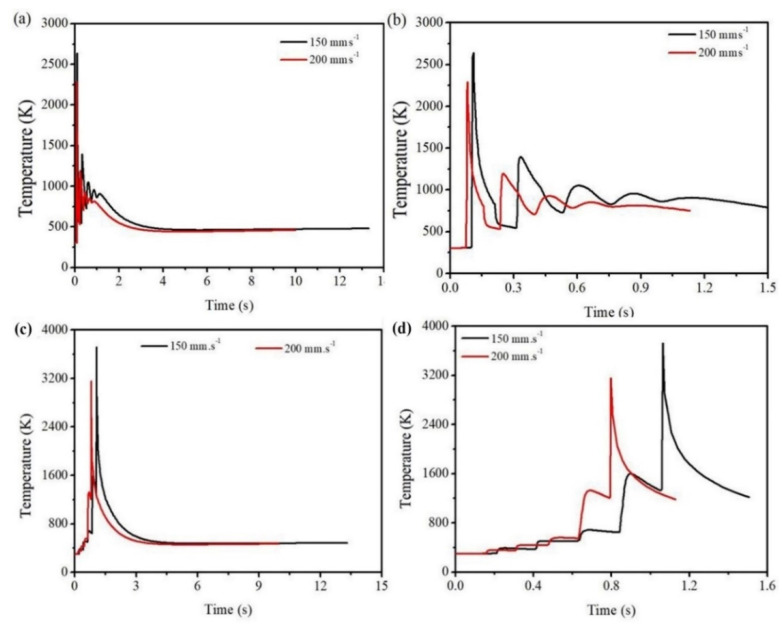
Time-temperature history (**a**) full view, (**b**) zoomed view at the centre of 1st layer, (**c**) full view, (**d**) zoomed view at the end of 5th layer at 200 W laser power and at a scan speed of 150 and 200 mm·s^−1^.

Theoretically, there should be five and four decreasing peak temperatures corresponding to the 1st and 2nd layer. Similarly, three, two, and one peak temperature corresponding to the 3rd layer, 4th layer and 5th layer are observed. The same phenomenon is also observed in the present case, and it can be identified from Figure 7a,b. However, Figure 6 has only 4 visible peaks due to low laser power. This shows that when the laser is in the 5th layer, the temperature at the 1st layer hardly has any effect. However, there is a rapid decrease in temperature after the laser is applied. After the rapid decline in the temperature, it is gradually reduced as cooling takes place.

### 3.2. Effects of Scanning Speed on the Melt-Pool and Temperature Distribution

As the laser scan speed increases with constant laser power, the depth as well as the width of the melt-pool decreases. Time-temperature history in the middle of the 1st layer is shown in Figure 7a,b. It can be identified that at a scan speed of 150 mm·s^−1^, a maximum temperature of 2640 K is attained, while at a scan speed of 200 mm·s^−1^, the highest temperature is 2287 K. Similar trend is also observed in the case of 5th layer (Figure 7c,d). At a scan speed of 150 mm·s^−1^, the maximum peak temperature is 3722 K, while at a scan speed of 200 mm·s^−1^, the peak temperature is 3154 K. This indicates that the current LAM machines have the capability to produce high layer thickness parts.

**Figure 8:**
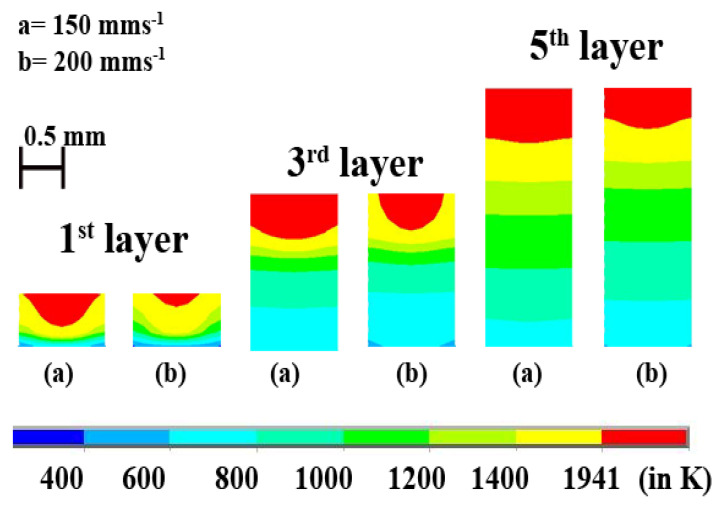
Changing melt-pool shapes for the changing scanning speed for the constant power source of 200 W and scanning speed of (**a**) 150 mm·s^−1^ and (**b**) 200 mm·s^−1^.

Figure 8 shows the decreasing depth of melt-pool corresponding to the 1st layer, 3rd layer, and 5th layer at different scan speeds. The depth of the melt-pool decreases from ~0.31 to ~0.12 mm for 1st layer, from ~0.45 mm to ~0.36 mm for the 3rd layer, and for the 5th layer, it decreases from ~0.55 to ~0.41 mm. Similarly, the width of the melt-pool decreases from ~0.87 to ~0.5 mm for the 1st layer, from ~1 to ~0.7 mm, for the 5th layer. It is evident that for both the processing conditions, melting of the powder material over the whole layer thickness is incomplete in the 1st layer. However, subsequent layers are fully melted. Moreover, a further study on increasing layer thickness will provide extensive information to determine the optimal parameters.

### 3.3. Effects of Laser Power on the Melt-Pool and Temperature Distribution for Pure Ti

**Figure 9:**
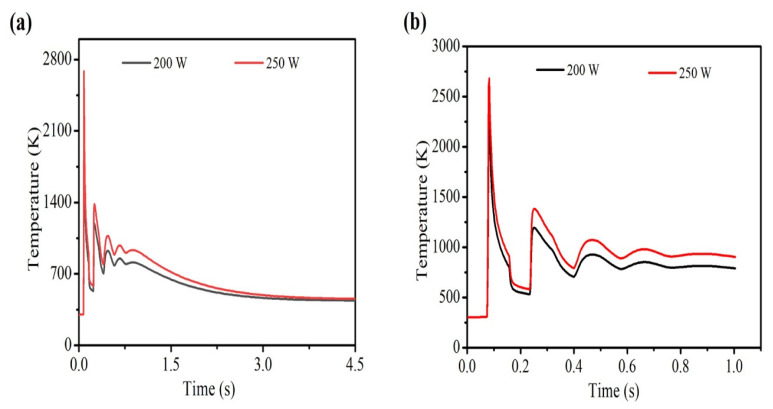
Effect of laser power in the middle of the 1st layer when laser power is 200, 250 W and scan speed is 200 mm·s^−1^ (**a**) full view and (**b**) magnified view.

**Figure 10:**
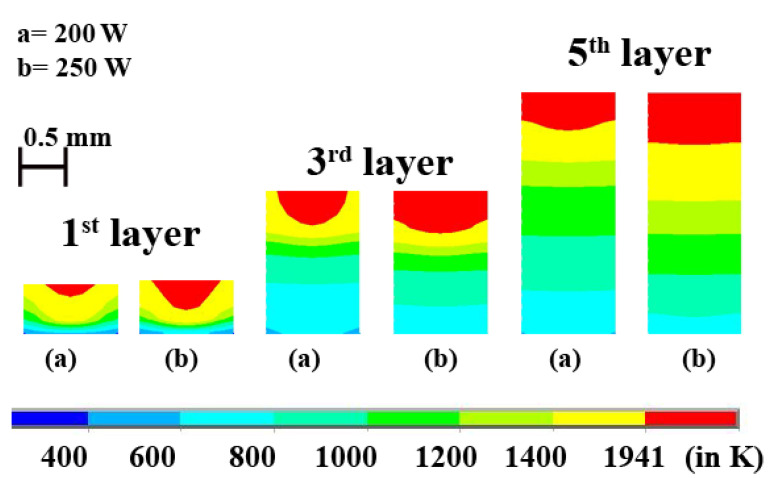
Evolution of melt pool dimensions for (**a**) *P* = 200, and (**b**) 250 W, *v* = 200 mm·s^−1^ in the 1st, 3rd and 5th layer.

Laser power is directly proportional to the temperature at any point during the LAM process, provided all other parameters are kept constant. Figure 9 shows the increasing final maximum temperature for increasing laser power at constant laser scan speed. The temperature variation at different laser power and at a constant scan speed of 200 mm·s^−1^ is shown in Figure 9. As discussed above, during the process, there are five major peaks in the 1st layer. The maximum temperature obtained at the centre of the 1st layer is 2288 K when laser power is 200 W and 2685 K when laser power is 250 W. Figure 10 represents the difference in melt-pool profiles corresponding to different laser power and at a constant scanning speed of 200 mm·s^−1^. A similar trend of Figure 7 is observed in Figure 9 wherein, 1st layer is not fully melted. However, as the process continues with new layers are being deposited, the temperature keeps increasing. Thereby, a fully melted pool is obtained across the whole cross-section of the layer.

The decreasing nature of peaks represents the gradual decrease of the impacts of laser power on the preceding layers as new layers are being deposited. In Figure 9b, the 5th peak has the lowest temperature in the 1st layer, and it is just above the temperature before cooling starts. This shows the negligible significance of laser in the 5th layer on the maximum temperature in the 1st layer. Figure 9 has overlapping peaks, but their peak values are different since both have the same scanning speed and different laser power. Figure 10 displays the evolution of melt-pool shape as new layers are added. It is evident that the width of the melt-pool increases faster than the depth of the melt-pool. This is due to the larger surface width area interaction with the laser. The increase in depth of melt-pool is significant and should not be discarded.

### 3.4. Comparative Study of Pure Ti and Inconel 718 Alloy

The pure Ti and Inconel 718 have different mechanical and thermal properties. It is self-evident that the melt-pool dimensions and temperature distribution of the two materials will be different for the same process parameters. Since the main influencing factors viz. laser power and laser scanning speed are the same for the two materials, a quantitative analysis is performed. Such an analysis will give more details about the intensity of temperature distribution and the melt-pool nature. This helps in choosing the right material under different circumstances where cost-saving, or safety may be the driving factor. It will be quite beneficial when multi-material manufacturing is being carried out and the difference in temperature and melt-pool becomes very important for the two materials to be fused in a homogenous way. This is especially very interesting concerning the printing of functionally graded materials. In such cases, there is a need to know if the different materials will melt and interact at the time at a given set of process parameters.

**Figure 11:**
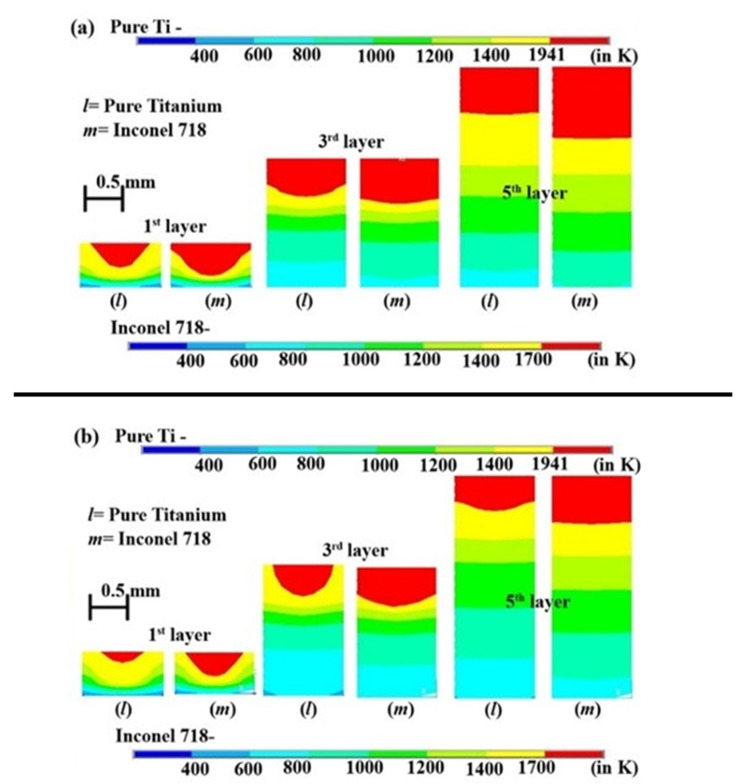
The difference of melt-pool dimensions for pure Ti and Inconel 718 alloy for the (**a**) dataset#4 and (**b**) dataset#3.

It is observed that the pure Ti material has a lower temperature and melt-pool dimensions than the Inconel 718 alloy for the same process parameters. It indicates that Inconel 718 requires lesser laser power to attain the same temperature as pure Ti material. Figure 11a shows the cross-sectional view of the variation of melt-pool of the two materials at 1st layer, 3rd layer, and 5th layer when the laser power is 250 W and laser scanning speed is 200 mm·s^−1^. The difference in the melt-pool dimensions can be attributed to the difference in material properties. From Figure 2, it is observed that the Inconel 718 has higher thermal conductivity as temperature increases while pure Ti has higher specific heat as the temperature increases. This shows that the specific heat outweighs the thermal conductivity whenever the maximum temperature of a layer is concerned. Higher specific heat means a longer time to attain a higher temperature. The melt-pool depths for the first layer are ~0.28 and ~0.38 mm for pure Ti and Inconel 718 alloy, respectively. For the third layer, the melt-pool depths are ~0.46 and ~0.54 mm for pure Ti and Inconel 718 alloy, respectively. The melt-pool shape of the fifth layer is almost the same as flat for both cases. However, there is a difference in the melt-pool thickness, which is ~0.56 and ~0.87 mm for pure Ti and Inconel 718.

Figure 11b represents the difference of melt-pool dimensions between pure Ti and Inconel 718 alloy when laser intensity is 200 W, and scan speed is 200 mm·s^−1^. The depth of melt-pool in the first layer is ~0.14 and ~0.28 mm for pure Ti and Inconel 718 alloy, respectively. For the third layer, the depth of the melt-pool is ~0.38 and ~0.47 mm for Ti and Inconel 718, respectively. In the 5th layer, the depth of melt-pool is ~0.42 and ~0.59 mm for Ti and Inconel 718 alloy, respectively.

**Figure 12:**
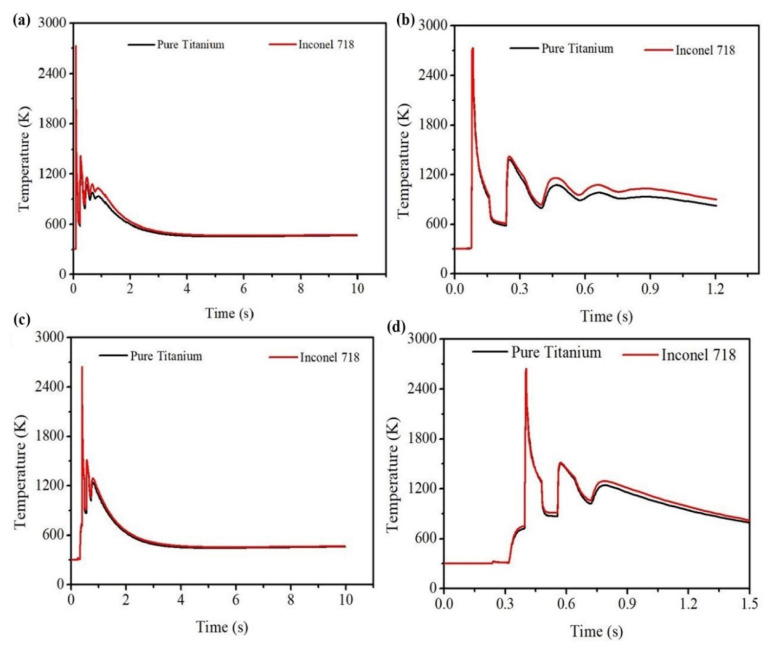
Time-temperature history of pure Ti and Inconel 718 alloy (**a**,**b**) at the centre of the 1st layer for dataset#4 and (**c**,**d**) at the centre of the 3rd layer for dataset#3.

Figure 12a,b shows the corresponding time-temperature history. Figure 12a is the time-temperature history considering the whole manufacturing time, while Figure 12b is the magnified view where peak temperature is represented. The maximum temperatures achieved by the laser power at the centre of the 1st layer are 2684 and 2732 K for pure Ti and Inconel 718, respectively. Figure 12c,d shows the temperature profile at the middle point of the 3rd layer over time. A similar trend is found in this case also. The peak temperature attained at the centre of the third layer in the case of pure Ti is 2599 K, while for Inconel 718 alloy, the peak temperature is 2644 K. Another interesting observation is the nature of the changing shape of melt-pool for both materials as the process continues. At first, the cross-sectional melt-pool shape is semi-circular, but it gradually changes to the shape of a ship’s hull. This shows that the melt-pool width has a higher sensitivity of change than that of the melt-pool depth.

## 4. Conclusions

A 3D finite-element model of laser-based additive manufacturing is developed to investigate melt-pool complexity and temperature behavior during the laser application throughout the model. One of the peculiar features of the model is the consideration of a high layer thickness as opposed to the general consideration of very thin layer thickness. The five-layer FEM simulation is performed to understand the effects of laser power and laser scan velocity on heat flow, melt-pool shape, and temperature distribution of the Ti and Inconel 718 materials. With the current study, the following conclusions can be drawn:

- For enough laser power and laser scan speed, LAM for a high layer thickness is possible for both Ti and Inconel 718 alloy materials. The current technology specifications have the capability to perform it.
- The temperature of subsequent layers increases as the process continues, and the number of layers increases. During the initial layers, the substrate is the primary agent for the heat transfer away from the layers.
- At the initial stage, there are parts of the layer that are not fully melted. However, this changes as the process continues and the number of layers increases.
- For low laser power, the impacts of laser power become very low in the 1st layer as the laser is acting on the 5th layer. For high laser power, there is a significant rise in overall temperature in the 1st layer even if the laser is scanning at the top layer.
- The melt-pool and maximum peak temperature are increased when the laser power is increased while the laser scanning velocity is kept constant for each material. However, the melt-pool dimensions and temperature are decreased when the scan velocity is increased while keeping a constant laser power.
- It is also found that the pure Ti has a lower peak temperature and melt-pool dimensions than the Inconel 718 alloy for the same influencing parameters, such as laser power and laser scan velocity, due to the difference in the material properties.
- There is a rapid change in the melt-pool shape of Inconel 718 from a semi-circle to a convex hull shape as the process continues.
- Further research of the current model can be implemented with the mechanical analysis to study the mechanical properties and residual stress analysis. Additionally, this model can be used as a base model to extend the research to a multi-track, multi-layer model during a laser-based additive manufacturing process.

## Figures and Tables

**Table 1:** LAM processing parameters for pure Ti Inconel 718 material.

**Data Set No.**	1	2	3	4	5	6
**Speed (mm/s)**	150	150	200	200	300	300
**Power (W)**	200	250	200	250	200	250

**Table 2:** Material properties of Ti and Inconel 718 used in the present work [61,62,63].

Parameter	Material		Unit
	Inconel 718	Pure Ti	
Density	8190	4.51 × 10^3^	Kg·m^−3^
Thermal conductivity	11.4	20	W·m^−1^·K^−1^
Melting point	1609–1700	1941	K
Specific heat	435	518	J·kg^−1^·K^−1^
Latent heat	152 × 10^3^	292 × 10^3^	J·kg^−1^
Co-efficient of thermal expansion	1.3 × 10^−5^	2.09 × 10^−5^	K^−1^

## Data Availability

The data presented in this study are available on request from the corresponding author.
